# Health and Disease in Relation to Marriage and the Married State

**Published:** 1904-12

**Authors:** 


### Reviews of Books.

**Health and Disease in Relation to Marriage and the Married State** Edited by Dr. H. SENATOR and Dr. S. KAMINER. Translated by J. DULBERG, M.D. Vol. I. Pp. 498. New York and London: Rebman Ltd. 1904.

This work is a translation by Dr. Joseph Dulberg, of Manchester, from the German edition. It consists of a series of contributions by various German physicians, several of whom, as Moll and Eulenberg, are well known amongst us. It treats of the relation of disease in the male and female with regard to marriage: what effect the physical state of husband and wife has or may have upon each other by their union, as well as upon their descendants, and indeed the welfare of whole families and communities.

The procreative faculties are dwelt upon with a plainness which seems hardly essential or in keeping with that English modesty which does not care to enter into the inner penetralia of married life, and which perhaps is more in keeping with Hindu ideas as displayed in the Sanskrit work of the "Ananga-Ranga."

No doubt greater care is requisite that the physical conditions of the contracting parties should be taken into account in proposed marriages, and the medical profession has already done much to guide the public aright in this matter, and might do more and so prevent a vast amount of misery. The more the medical profession becomes acquainted with the conditions bearing on the subject no doubt so much the more will an advance in this direction take place. It is the method to be adopted and made absolute that is the difficulty.

Still there can be little doubt that if the contents of this work were to be put into actual practice, men and women who are desirous to marry would all have to be entered on a kind of matrimonial register, giving a correct description of all their mental and bodily qualifications and defects for such union, and that on oath. Only in this way could all the great risks and sad events we so often now become acquainted with as an outcome of unfitting alliances between male and female be in any degree done away.

Much of course must rest upon the advice of the medical adviser. As has been said not long ago[1]: "Many a man who requires a bracing obtains a relaxing wife, and many a woman who requires a relaxing secures a bracing husband. The matrimonial adviser, when consulted, might prescibe as follows: 'Sir, you are a weak-willed man, and it is necessary for you to marry a bracing woman who will rule you with a firm but gentle hand. Were you united to a relaxing woman you would probably be wretched.'"

In this volume is a series of papers collected into a compact body on a number of matters and questions dealing with the ante-nuptial, the nuptial and post-nuptial states. Amongst these subjects one finds discussed the hygiene of marriage, consanguinity, climatic influence, menstruation, diseases of organs and such-like matters. Vol. II., which is to follow, will deal with sexual diseases, diseases of women, insanity, intoxications, &c., &c. Much of the matter is interesting and of a useful character.

